# Between inclusion and risk: participation in online gambling and cognitive distortions in young people with motor disabilities

**DOI:** 10.3389/fpsyg.2026.1859781

**Published:** 2026-07-22

**Authors:** Raquel Suriá-Martínez, Fernando García-Castillo, Carmen López-Sánchez, José A. García del Castillo

**Affiliations:** 1 Department of Communication and Social Psychology, University of Alicante, Alicante, Spain; 2 Department of Education, University of Alicante, Alicante, Spain; 3 Department of Health Psychology, Miguel Hernández University ofElche, Alicante, Spain

**Keywords:** cognitive distortions, motor disability, online gambling, problem gambling, university students

## Abstract

This study examines participation in online gambling, explores the relationship between cognitive distortions (gambler’s fallacy and illusion of control) and gambling severity, and evaluates their predictive role in the risk of problem gambling. A cross-sectional study was conducted with a sample of 135 young people with motor disabilities from major Spanish disability associations. Questionnaires were used to assess participation in online gambling, cognitive distortions, and level of gambling risk. Descriptive, correlational, logistic regression analyses, and a mediation model with bootstrap were performed. A total of 60.7% of participants reported having engaged in online gambling. Cognitive distortions were significantly associated with gambling severity. Logistic regression indicated that both dimensions predict gambling risk (OR = 1.21–1.24, *p* < 0.01), with an AUC of 0.81. The mediation model showed that cognitive distortions partially mediate the relationship between participation in online gambling and gambling risk. The results suggest that cognitive distortions play a key role in explaining gambling risk among students with motor disabilities, representing a relevant target for prevention and intervention.

## Introduction

Gambling has historically been a popular form of entertainment, ranging from sports betting to traditional casinos. However, gambling disorder is recognized as a clinical condition associated with significant psychological, social, and financial consequences. According to the DSM-5 and the ICD-11, Gambling Disorder is classified as a behavioral addiction characterized by persistent and recurrent problematic gambling behavior that leads to clinically significant impairment or distress (Girone et al., 2026; Lombardi et al., 2024; Tulloch et al., 2026). These consequences include financial difficulties, interpersonal conflicts, emotional distress, anxiety, and depressive symptoms, as well as functional impairment in academic and occupational domains (Forrest et al., 2026; Oyanedel et al., 2024; Samaniego et al., 2025).

From this clinical perspective, the development of online gambling environments has intensified concerns regarding accessibility and risk exposure. The development of the internet and digital platforms has profoundly transformed access to gambling activities, allowing users to participate at any time and from virtually anywhere (Fiedor et al., 2025; Ji et al., 2024; Vepsäläinen et al., 2024). This process of digitalization has contributed to significant growth in online gambling (i.e., gambling activities such as online sports betting, online casino games, poker, and lotteries, explicitly distinct from video gaming or sports-related activities) in recent years.

In this context, young people have become one of the groups most exposed to this phenomenon. University students, in particular, represent a potentially vulnerable group to problematic online gambling due to various factors such as academic stress, social pressure, the search for new experiences, or the expectation of obtaining quick financial gains (Angelini et al., 2024; Fraiwan and Almomani, 2025; Fiedor et al., 2025; Tulloch et al., 2026). Furthermore, the university context itself involves intensive use of the internet and technological devices for academic and social activities, increasing exposure to digital environments where multiple online gambling platforms (sports betting, casino games, poker, lotteries, etc., distinct from video game platforms) are available. Within this group, certain populations may present additional vulnerability, such as students with disabilities (Osuna et al., 2023; Pitt et al., 2021; Suriá-Martínez et al., 2024a,b). In particular, students with motor disabilities may face physical barriers or mobility difficulties that limit their access to certain in-person or traditional leisure spaces. These limitations are not only physical in nature but may also be associated with psychosocial factors such as social isolation, reduced opportunities for face-to-face interaction, and lower participation in conventional recreational activities, which in turn increase emotional vulnerability and the search for accessible leisure alternatives.

In this regard, the literature has shown that people with disabilities may experience higher levels of perceived loneliness and restrictions in social participation, factors that are associated with greater compensatory use of digital environments as a form of interaction and coping (Badia et al., 2013; Emerson et al., 2021). This pattern can be understood from the social exclusion model, according to which limitations in community participation increase the likelihood of resorting to alternative environments that provide immediate gratification or a sense of belonging (Stancliffe and Hall, 2023).

As a consequence, the digital environment can become a particularly accessible alternative for leisure and social interaction. However, this increased availability may also involve greater exposure to potentially problematic behaviors, such as excessive internet use or participation in risky activities, including online gambling (Suriá-Martínez et al., 2024a,b; Kuss and Griffiths, 2017). In this context, internet use has been proposed to serve functions of emotional regulation and experiential escape, especially in individuals with higher levels of social isolation or psychological distress (Caplan, 2003; Kardefelt-Winther, 2014).

From this perspective, online gambling may act as a maladaptive coping strategy in response to negative emotional states, loneliness, or frustration derived from functional limitations, as it provides immediate stimulation, a sense of control, and intermittent reinforcement. These mechanisms have previously been associated with a higher risk of behavioral addictions in vulnerable populations (Brand et al., 2019; Dong and Potenza, 2014).

In this sense, several studies have highlighted that the internet and digital technologies are important tools for facilitating the social, educational, and recreational participation of people with disabilities (Chia and Zhang, 2020; Pallesen et al., 2021; Pitt et al., 2021; Solish et al., 2010), although they may also entail new risks when used as the primary means of emotional regulation or as compensation for deficits in face-to-face social interaction (Suriá-Martínez et al., 2024a,b). In the case of online gambling, characteristics such as constant accessibility, anonymity, and immediacy of rewards may favor the development of problematic gambling patterns in certain users.

In Spain, participation in online gambling has experienced notable growth in recent years. According to reports from the Directorate General for the Regulation of Gambling (DGOJ), approximately 7.2% of the adult population participated in some form of online gambling during the past year. This increase has raised growing concern about the potential risks associated with the development of problem gambling behaviors and addiction.

From an economic and behavioral perspective, gambling can be understood as a decision-making activity in contexts of uncertainty and risk. However, various approaches within behavioral economics have shown that these decisions do not always follow strictly rational criteria but are mediated by cognitive biases, heuristics, and emotional factors. Within this framework, certain types of online gambling appear to encourage greater participation and higher risk of addiction (Hautamäki et al., 2025).

Unlike more traditional forms such as in-person poker or roulette in physical settings, these online gambling formats tend to generate greater time engagement and higher participation frequency due to constant availability of events, immediacy of bets or plays, the possibility of performing multiple actions in short periods, and continuous integration of information from experts, statistics, or automated game dynamics. This combination reinforces player involvement and may increase the perception of control over outcomes that ultimately remain uncertain.

In this regard, the literature suggests that this type of behavior can be partly explained through prospect theory proposed by Kahneman and Tversky (1979), according to which individuals evaluate gains and losses asymmetrically and tend to overestimate the probability of rare events. This bias leads to a distorted perception of risk and expected benefit, which may encourage participation in high-risk activities such as online gambling (including sports betting and casino games). Consequently, the interaction between these cognitive biases and the structural characteristics of online gambling platforms, such as constant accessibility, rapid rewards, and continuous stimulation, contributes to increasing the likelihood of developing intensive or problematic gambling patterns, especially in contexts where immediacy and ease of access facilitate repetitive behavior.

Within this framework, cognitive distortions play a fundamental role in understanding gambling behavior. These distortions can be defined as erroneous patterns of thinking that influence the interpretation of gambling outcomes and the player’s decision-making (Kolberg et al., 2025; Samaniego et al., 2025; Solé et al., 2023). Among the most common cognitive distortions in gambling are the gambler’s fallacy, which involves the belief that past outcomes influence future results; unrealistic optimism, characterized by the tendency to overestimate the probability of winning; and the illusion of control, which consists of believing that one can influence outcomes that depend on chance (Chan et al., 2019; Ruiz and López, 2016).

The role of these cognitive distortions has been widely explained through the cognitive model of problem gambling proposed by Ladouceur et al. (2002). According to this model, problem gambling behaviors are largely maintained by erroneous beliefs about how chance and probabilities operate. These beliefs can reinforce persistence in gambling even after repeated losses, contributing to the development of addictive behavioral patterns. Although the relationship between youth, online gambling, and cognitive distortions has been widely studied, there is still limited attention to specific groups within the university context, such as students with motor disabilities. Analyzing gambling participation patterns and cognitive distortions in this group is relevant for better understanding the factors that may contribute to the development of problematic gambling behaviors, as well as for designing prevention and intervention strategies tailored to their needs.

The main purpose of this study was to analyze participation in online gambling and its relationship with gambling risk among university students with motor disabilities, as well as to explore the role of cognitive distortions in the severity and prediction of problematic gambling behaviors.

*Objective 1*: Examine participation in online gambling among university students with motor disabilities, identifying the most frequent gambling modalities and the distribution of gambling risk profiles (no risk, at risk, and problem gambling).

*Objective 2*: Analyze whether there are significant differences in gambling risk profiles according to the type of modality practiced (sports betting, online casino, online poker, online lotteries).

*Objective 3*: Evaluate the relationship between gambling-related cognitive distortions (gambler’s fallacy and illusion of control) and gambling severity, comparing these distortions across different risk profiles.

*Objective 4*: Determine the predictive role of cognitive distortions in the likelihood of presenting problematic gambling behaviors.

Based on these objectives, the following hypotheses were proposed:

*H1*. Participation in online gambling will be frequent among university students with motor disabilities, with modalities such as sports betting and online casino games predominating.

*H2*. Risk profiles will differ significantly depending on the type of gambling modality practiced, with sports betting and online casino games being associated with higher gambling risk.

*H3*. Gambling-related cognitive distortions (gambler’s fallacy and illusion of control) will progressively increase as the level of gambling risk rises.

*H4*. Cognitive distortions (gambler’s fallacy and illusion of control) will significantly predict the likelihood of problematic gambling behaviors, showing a model with good discriminative capacity and statistical fit.

## Method

### Participants

The sample consisted of 135 young adults with motor disabilities, all over 18 years of age, recruited through major Spanish organizations and platforms that provide services to people with physical disabilities. Eligibility was limited to participants holding an official disability certificate recognized by the Spanish Social Security system; individuals without such certification were excluded.

To facilitate participant recruitment, meetings were held with representatives of the main organizations in the field of motor disabilities in order to obtain institutional consent and their collaboration in the dissemination and administration of the questionnaires.

Participants presented different types of motor disabilities, including cerebral palsy (22%), spinal cord injury (28%), spinal muscular atrophy (14%), muscular dystrophies (11%), and other congenital or acquired physical conditions (25%). All participants had official certification confirming their disability status.

Regarding sociodemographic characteristics, most participants had completed primary education (65%), 35% had completed secondary education, and only a small proportion had attained higher education. In terms of employment status, the majority were unemployed, receiving disability benefits, or actively seeking employment. Participants were between 18 and 35 years old (M = 24.1, SD = 4.6), with a gender distribution of 53% male and 47% female. Most participants resided in urban areas (65%), while the remainder lived in semi-urban settings (35%).

Participation was voluntary and anonymous. All participants received prior information regarding the study objectives and data confidentiality. The study was conducted in accordance with the principles of the Declaration of Helsinki and the General Data Protection Regulation (EU 2016/679). Written informed consent was obtained from all participants, and the study protocol was approved by the university’s Ethics Committee.

### Instruments

The Massachusetts Gambling Screen (MAGS) (Shaffer et al., 1994) is a 19-item screening instrument designed to assess gambling behaviors and detect potential addiction-related problems. It evaluates dimensions of gambling behavior, such as the frequency and patterns of both online and offline gambling, and has previously reported good internal consistency (*α* = 0.82). The original instrument includes both dichotomous (yes/no) and Likert-type response formats and classifies participants into three categories: no gambling problems (<3), moderate problems (3–5), and severe or probable pathological gambling (≥6).

In the present study, a Spanish-adapted version of the MAGS was used, incorporating items specifically related to online gambling and internet-based betting. This adaptation was carried out by the authors and included a translation and back-translation review process to ensure conceptual and linguistic equivalence with the original version. The selection of this instrument over other problem gambling scales is justified by its brevity, its applicability in non-clinical contexts, and its capacity to jointly assess online and offline gambling behaviors, making it particularly suitable for populations that may present functional limitations, such as individuals with motor disabilities.

Furthermore, the items were adapted into a five-point Likert-type response format in order to improve measurement sensitivity, allowing for a more nuanced and gradual assessment of gambling frequency and severity. This modification was introduced to reduce the limitations associated with dichotomous response formats and to better capture behavioral variability in the target population.

To examine the psychometric properties of the adapted instrument, a pilot sample of 62 participants was used to conduct a confirmatory factor analysis (CFA). Prior to the CFA, statistical assumptions were assessed, including sampling adequacy (KMO ≥ 0.60), Bartlett’s test of sphericity (*p* < 0.05), inter-item correlations, distributional assumptions (skewness and kurtosis), and the absence of extreme outliers. The CFA confirmed the factorial structure of the instrument, with factor loadings above 0.60 and satisfactory model fit indices. Finally, internal consistency in the study sample was adequate (*α* = 0.86), supporting the reliability of the adapted version of the MAGS.

To assess gambling-related cognitive distortions, the *Gambling Beliefs Questionnaire* (GBQ; Steenbergh et al., 2002) was used in its Spanish-adapted version developed by Suriá-Martínez et al. (2024a,b). This version was selected due to its adequate psychometric properties obtained in young and university populations, as well as the cultural and linguistic appropriateness of the adaptation. The instrument assesses gambling-related cognitive distortions through 20 items rated on a 5-point Likert scale (1 = not at all to 5 = extremely).

The original instrument shows high internal consistency (*α* = 0.92) and adequate test–retest reliability (*r* = 0.77). It is composed of two factors: (a) Luck/Perseverance (*α* = 0.90), which reflects beliefs about the role of luck and persistence in gambling outcomes, and (b) Illusion of Control (*α* = 0.84), which reflects perceived control over chance-based outcomes. The Spanish adaptation confirmed the bifactorial structure of the instrument and demonstrated adequate validity and reliability indices.

In the present study, the same factorial structure was retained, with factor loadings above 0.60 for all items. Likewise, internal consistency indices were high: *α* = 0.90 for the Luck/Perseverance dimension, *α* = 0.84 for Illusion of Control, and *α* = 0.98 for the total scale, indicating excellent reliability of the instrument in the analyzed sample.

### Procedure

Data were collected during the 2024-2025 academic year using self-administered questionnaires available in both paper and digital formats (via accessible Google Forms), allowing participants to select the format best suited to their needs. Recruitment was conducted through disability associations and support networks in the Valencian Community.

The inclusion criterion was having accessed online gambling or sports betting at least once during the past year; all other participants were excluded from the study. Individuals with intellectual disabilities or medical conditions that impaired comprehension of the questionnaires were excluded.

Data collection was completed in a single session lasting approximately 30–35 min. Participants received detailed information about the study and provided written informed consent prior to participation. Accessibility accommodations were provided when necessary, including extended response time or assistance with questionnaire completion. Upon completion, participants were provided with information on mental health resources and responsible gambling support services.

### Data analysis

Data analyses were conducted using SPSS version 28 and Hayes’ PROCESS macro for mediation analyses. Statistical significance was set at *p* < 0.05. Assumptions of normality, homogeneity of variance, and absence of outliers were verified prior to analysis.

For Objective 1, descriptive analyses were conducted to examine participation in online gambling modalities (sports betting, online casino, online poker, and lotteries) and their distribution across gambling risk profiles. Results were presented as frequencies and percentages.

For Objective 2, differences in risk profiles across gambling modalities were examined using chi-square tests of independence. *Post hoc* analyses were conducted using standardized residuals to identify significant group differences.

For Objective 3, means and standard deviations of cognitive distortions (gambler’s fallacy and illusion of control) were computed across risk groups. One-way ANOVAs were conducted to assess group differences, with *F*-values, degrees of freedom, *p*-values, and partial eta squared (*η*^2^) reported. Tukey *post hoc* tests were used for pairwise comparisons. Pearson correlation coefficients were calculated to examine associations between cognitive distortions and gambling severity.

For Objective 4, binary logistic regression analyses were performed to assess the predictive role of cognitive distortions. The dependent variable was recoded as 0 = no risk and 1 = at risk or problem gambling. Model fit was evaluated using the model *χ*^2^ statistic, Nagelkerke *R*^2^, classification accuracy, and the Hosmer–Lemeshow test. Discriminative ability was assessed via ROC curve analysis (AUC with 95% confidence intervals). Additionally, a partial mediation analysis using bootstrapping (5,000 samples) was conducted to examine whether gambling severity mediated the relationship between cognitive distortions and gambling risk.

## Results

### Objective 1: participation in online gambling and risk profiles

Participation in online gambling was examined in 135 university students with motor disabilities, as well as its relationship with gambling risk profiles. The results showed that 82 students (60.7%) had participated in at least one form of online gambling in the past year, while 53 students (39.3%) reported no participation.

Among the most frequent modalities, sports betting was the most reported (46 students, 34.1%), followed by online casino games (29 students, 21.5%), online lotteries (24 students, 17.8%), and online poker (19 students, 19.3%).

Regarding risk profiles, the majority of gambling participants were classified as having no gambling problems (125 cases across modalities), while 28 participants (16.1%) were classified as presenting moderate problems and 21 participants (12.1%) showed severe or probable pathological gambling. These distributions indicate variability across gambling modalities, with higher proportions of moderate and severe or probable pathological gambling observed among sports betting and online casino participants (Table 1).

**Table 1:** Participation in online gambling modalities and risk profiles.

Gambling modality	No gambling problems *n* (%)	Moderate problems *n* (%)	Severe or probable pathological gambling *n* (%)	Total *n* (%)
Sports betting	28 (60.9)	12 (26.1)	6 (13.0)	46 (34.1)
Online casino	18 (62.1)	6 (20.7)	5 (17.2)	29 (21.5)
Online poker	10 (50.0)	5 (25.0)	5 (25.0)	20 (14.8)
Online lotteries	16 (61.5)	5 (19.2)	5 (19.2)	26 (19.3)
Did not participate during the last month	53 (100)	0 (0)	0 (0)	53 (39.3)
Total	125	28	21	174

Participants could select more than one modality.

### Objective 2: differences in risk profiles by type of gambling

To examine whether there were differences in risk profiles according to the type of online gambling activity, a chi-square test of independence was conducted between gambling modality (sports betting, online casino, online poker, and online lotteries) and gambling severity level (no problems, at-risk, and problem gambling). The results showed a statistically significant association between the two variables, *χ*^2^ (6) = 12.87, *p* = 0.045, indicating that the distribution of risk profiles was not homogeneous across gambling modalities.

Table 2 shows that online lotteries presented the highest proportion of participants with no gambling problems (66.7%) and the lowest proportion of problem gamblers (12.5%). In contrast, online casino gambling and sports betting showed relatively higher proportions of participants classified as at-risk or problem gamblers. In particular, online casino gambling recorded the highest percentage of problem gambling (17.2%), while online poker showed the highest proportion of at-risk participants (27.8%), although this modality exhibited an intermediate pattern overall compared to the other groups.

**Table 2:** Distribution of risk profiles by gambling modality and significant differences.

Gambling modality	No problems *n* (%)	At-risk *n* (%)	Problem gambling *n* (%)
Sports betting	27 (60.9%)ᵃ	12 (26.1%)ᵃ	7 (13.0%)ᵃ
Online casino	18 (62.1%)ᵃ	6 (20.7%)ᵃ	5 (17.2%)ᵃ
Online poker	10 (55.6%)ᵃᵇ	5 (27.8%)ᵃ	3 (16.6%)ᵃᵇ
Online lotteries	16 (66.7%)ᵇ	5 (20.8%)ᵇ	3 (12.5%)ᵇ
*χ*^2^ (6) = 12.87, *p* < 0.05			

Values with different superscript letters differ significantly according to *post hoc* analyses with Bonferroni correction (*p* < 0.05). Groups sharing at least one letter do not differ significantly from each other.

To identify the categories responsible for the observed overall association, *post hoc* analyses were conducted using adjusted standardized residuals and pairwise comparisons between gambling modalities, applying a Bonferroni correction to control for the increased Type I error associated with multiple testing. The results suggested that the most relevant differences were found between online lotteries and both sports betting and online casino gambling.

Specifically, participants engaged in online lotteries were significantly more likely to fall into the no-problem gambling category and significantly less likely to be classified as problem gamblers compared to users of sports betting and online casino gambling (*p* < 0.05). These differences are reflected in Table 2 through superscript letters assigned to each modality. Groups sharing the same letter do not differ significantly from each other, whereas groups with different letters show statistically significant differences.

Online poker, on the other hand, showed intermediate values and was labeled with the superscript “ab,” indicating that it did not differ significantly from either the groups labeled “a” or those labeled “b.” This pattern suggests that online poker occupies a transitional position between modalities associated with higher risk and those linked to less problematic gambling profiles.

Overall, the findings indicate that different types of online gambling may be associated with varying levels of vulnerability to the development of problematic gambling behaviors. While online lotteries were associated with lower-risk profiles, sports betting and online casino gambling showed a higher concentration of participants in the at-risk and problem gambling categories. Although these findings do not allow causal inferences due to the cross-sectional nature of the study, they provide evidence regarding the potential influence of structural characteristics of gambling modalities on the emergence and maintenance of problematic gambling behaviors. In particular, the high frequency of play, immediacy of rewards, and rapid betting cycles characteristic of sports betting and online casino gambling may contribute to an increased risk of developing maladaptive gambling patterns.

### Objective 3: cognitive distortions and gambling severity by risk profile

To examine the relationship between cognitive distortions and gambling severity, mean scores for gambler’s fallacy and illusion of control were first calculated according to risk profiles: no gambling problems, moderate problems, and severe or probable pathological gambling (Table 3).

**Table 3:** Cognitive distortions by player profile and ANOVA results.

Variable	Player profile	*M* ± SD	*F* (2,132)	*p*	Partial *η*^2^
Gambler’s fallacy	No gambling problems	1.8 ± 0.5	48.6	<0.001	0.424
	Moderate problems	2.6 ± 0.6			
	Severe or probable pathological gambling	3.4 ± 0.7			
Illusion of control	No gambling problems	1.9 ± 0.4	52.3	<0.001	0.442
	Moderate problems	2.7 ± 0.5			
	Severe or probable pathological gambling	3.5 ± 0.6			

Partial *η*^2^ indicates effect size; *M*, mean; SD, standard deviation.

The results showed a progressive increase in cognitive distortions as gambling risk level increased. For gambler’s fallacy, participants with no gambling problems had a mean of 1.8 (SD = 0.5), those with moderate problems had a mean of 2.6 (SD = 0.6), and participants with severe or probable pathological gambling showed the highest mean, 3.4 (SD = 0.7). Similarly, illusion of control showed means of 1.9 (SD = 0.4) for the group with no gambling problems, 2.7 (SD = 0.5) for those with moderate problems, and 3.5 (SD = 0.6) for participants with severe or probable pathological gambling.

ANOVA confirmed that these differences were statistically significant for both gambler’s fallacy (*F* (2, 132) = 48.6, *p* < 0.001, *η*^2^*_p_* = 0.424) and illusion of control (*F* (2, 132) = 52.3, *p* < 0.001, *η*^2^*_p_* = 0.442), indicating that approximately 42–44% of the variance in cognitive distortions is explained by risk profile. Tukey post-hoc tests showed that each group differed significantly from the others, revealing a linear pattern: higher gambling risk was associated with higher levels of cognitive distortions.

Additionally, Pearson correlations were conducted to assess the relationship between cognitive distortions and gambling severity across the entire sample (Table 4). The results showed positive and significant correlations. Gambler’s fallacy correlated with gambling severity at *r* = 0.54 (*p* < 0.01), while illusion of control showed a slightly higher correlation (*r* = 0.58, *p* < 0.01). Furthermore, the two cognitive distortions were strongly correlated with each other (*r* = 0.62, *p* < 0.01).

**Table 4:** Correlation matrix.

Variable	1	2	3
1. Gambler’s fallacy	—		
2. Illusion of control	0.62**	—	
3. Gambling severity	0.54**	0.58**	—

**p < 0.01.

### Objective 4: predictive role of cognitive distortions in gambling risk

With the aim of examining the predictive role of cognitive distortions in the risk of developing problematic gambling behaviors, a binary logistic regression analysis was conducted. The dependent variable was gambling risk profile, recoded into two categories: 0 = non-risk gambler and 1 = at-risk or problem gambler.

The overall model was statistically significant (*χ*^2^ = 31.62, d*f* = 2, *p* < 0.001), indicating that the inclusion of cognitive distortions significantly improves the model’s ability to explain the likelihood of belonging to the risk group compared to the non-risk group. In terms of overall fit, the model explained 38% of the variance in the dependent variable (Nagelkerke *R*^2^ = 0.38), suggesting a moderate-to-high explanatory power within behavioral research contexts. Additionally, the model demonstrated adequate classification accuracy, correctly classifying 76.3% of cases, supporting its practical utility in distinguishing between risk profiles.

Regarding predictive effects, logistic regression results showed that cognitive distortions significantly increased the likelihood of problematic gambling behavior. Overall, the model indicated that higher levels of cognitive distortions were associated with increased odds of belonging to the risk group, with an overall effect ranging from OR = 1.21 to OR = 1.24 (*p* < 0.05). This indicates that, for each unit increase in cognitive distortion scores, the odds of presenting gambling risk increase by approximately 21 to 24%, reinforcing their role as relevant vulnerability factors.

More specifically, mediation analyses showed that both gambler’s fallacy and illusion of control were significantly associated with gambling severity. Higher levels of these erroneous beliefs were related to greater severity of gambling behavior (*a*_1_ = 0.54, 95% CI [0.38, 0.68]; *a*_2_ = 0.58, 95% CI [0.43, 0.72]; *p* < 0.01), indicating that cognitive distortions not only predict risk status but are also associated with increasing behavioral severity.

In turn, gambling severity was significantly associated with the likelihood of belonging to the risk group (*b* = 0.31, 95% CI [0.19, 0.45]; *p* < 0.01), suggesting that severity acts as an intermediary mechanism linking cognitive distortions and final risk classification. In this sense, the results support an indirect pathway through which cognitive distortions influence gambling risk via increased behavioral severity.

However, the direct effect of gambler’s fallacy on gambling risk remained statistically significant (*c*′_1_ = 0.19, 95% CI [0.04, 0.37]; *p* < 0.05), indicating partial mediation. Thus, gambler’s fallacy influences gambling risk both indirectly, through severity, and directly, as an independent predictor.

**Figure 1:**
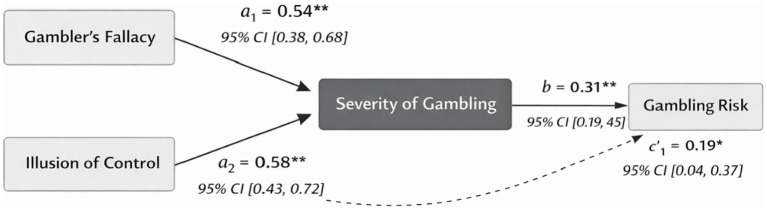
Partial mediation model of gambling risk.

Figure 1 illustrates this partial mediation model of gambling risk, showing both direct and indirect pathways among the studied variables.

**Figure 2:**
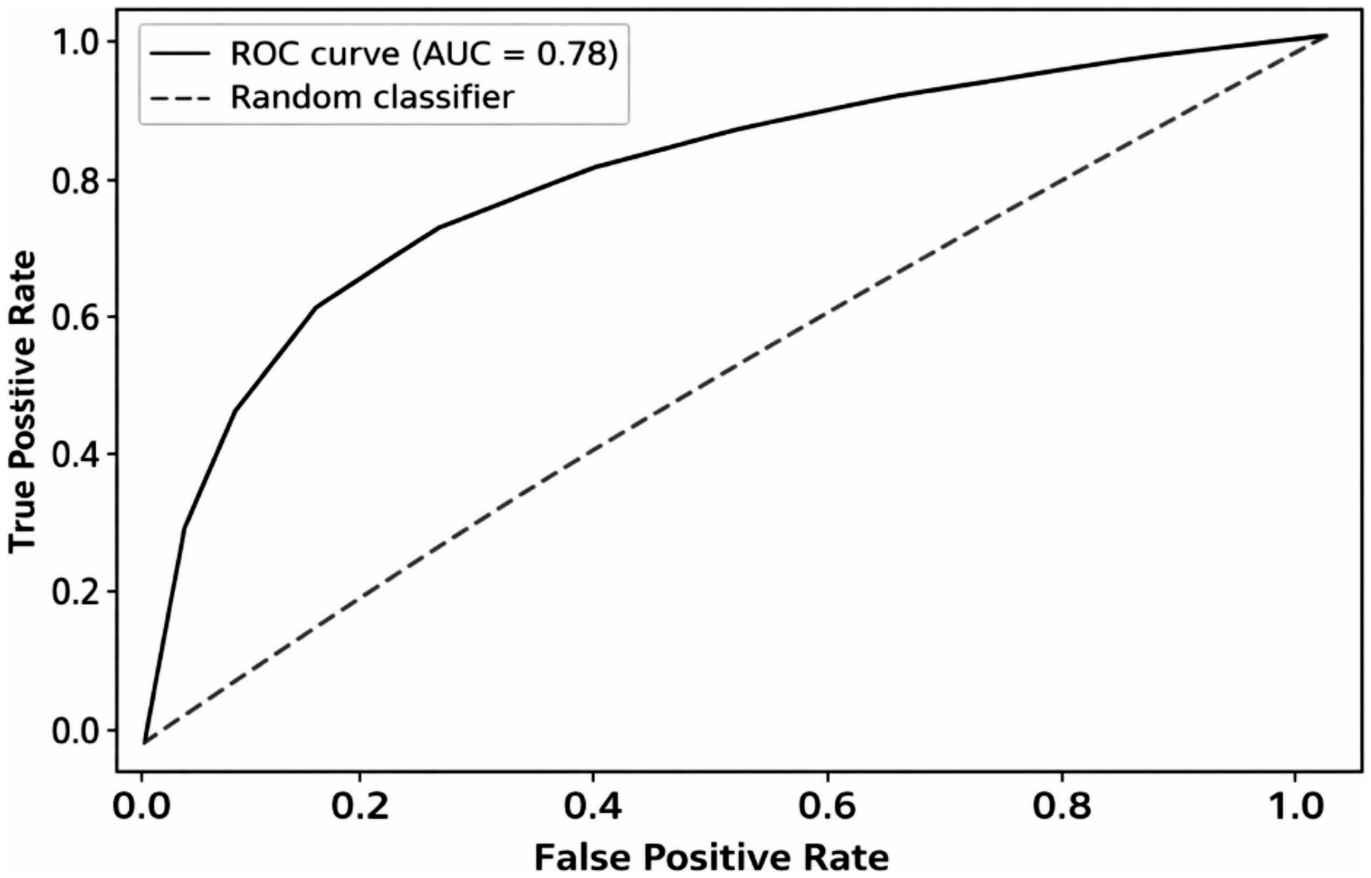
ROC curve for the logistic regression model predicting gambling risk.

Finally, regarding the discriminative ability of the model, ROC curve analysis yielded an area under the curve (AUC) of 0.81 (95% CI [0.73–0.88]), indicating good accuracy in distinguishing between risk and non-risk participants. This result reflects an appropriate balance between sensitivity and specificity, confirming the model’s utility not only statistically but also from an applied perspective for identifying at-risk profiles in this population (Figure 2).

## Discussion

This study provides evidence on the prevalence and patterns of online gambling (specifically online gambling activities such as sports betting, online casino games, and other gambling modalities, explicitly distinct from video gaming or gaming disorder behaviors), risk profiles, the relationship with cognitive distortions, and their predictive role among university students with motor disabilities.

In line with the first objective of the study (to analyze the prevalence and patterns of online gambling behavior), the findings showed that 60.7% of university students with motor disabilities reported having engaged in at least one gambling modality during the past year, with sports betting and online casino games being the most prevalent. (i.e., online gambling behaviors such as sports betting and online casino participation).

This finding is consistent with previous studies documenting high involvement of university students in online gambling, particularly in digitally accessible modalities with immediate rewards (Ghelfi et al., 2024; Girone et al., 2026; Osuna et al., 2023; Mancera et al., 2024; Sales and Cloquell, 2021). Likewise, the distribution of risk profiles indicated that most students were classified as non-risk gamblers, although there was a relevant subgroup of students at risk or with gambling problems, in line with Pilatti et al. (2020), who highlight the existence of vulnerable subpopulations in seemingly protected university environments. In this regard, Forrest et al. (2026) support that excessive participation in specific games, such as betting, can emerge even among individuals with disabilities, especially young people, becoming more attractive when accessible means and strong social and financial motivations are present.

Regarding the second objective (differences in risk profiles by type of gambling), the results showed that sports betting and online casino gambling were associated with higher proportions of students at risk or with gambling problems, whereas lotteries and online poker showed lower levels of risk. This pattern is consistent with evidence identifying certain types of gambling as more likely to induce problematic behaviors due to the frequency of outcomes and immediate feedback (Girone et al., 2026; Lombardi et al., 2024; Tulloch et al., 2026). Some studies have shown that sports betting and online casino players exhibit higher rates of problematic gambling compared to those participating in traditional lotteries, highlighting the importance of considering gambling modality as a risk factor in identifying vulnerable students (Frimpong and Acheampong, 2024; García-Aller et al., 2026; Pitt et al., 2021).

Regarding the third objective and hypotheses related to cognitive distortions, the results indicated a progressive increase in cognitive distortions as the level of risk increased. Non-risk students showed low mean levels of gambler’s fallacy and illusion of control, at-risk gamblers showed intermediate levels in both factors, and students with gambling problems exhibited the highest means in both cognitive distortions. ANOVA analyses confirmed statistically significant differences with considerable effect sizes across the different risk profiles.

These findings support the hypothesized role of cognitive distortions as central mechanisms in gambling severity and are consistent with the cognitive-behavioral framework of problem gambling (Ladouceur et al., 2002; Lombardi et al., 2024; Tulloch et al., 2026), which posits that cognitive distortions play a central role in the onset and maintenance of these behaviors. This model not only focuses on gambling behavior itself but emphasizes that how individuals interpret and think about gambling is the key element that sustains the problem. In this sense, authors such as Ruiz and López (2016) argue that gamblers develop erroneous beliefs about chance, such as illusion of control or biased reinterpretation of losses and gains, which contribute to maintaining gambling behavior. Similarly, Chan et al. (2019) highlight that these cognitive biases not only sustain behavior but also intensify it, making it more difficult to stop.

These findings are further supported by more recent research in specific populations, such as university students with disabilities. Studies such as Pitt et al. (2021) and Suriá-Martínez et al. (2024a,b) show that high levels of cognitive distortions are significantly associated with a greater risk of developing problem gambling. Overall, the literature suggests that dysfunctional beliefs about gambling are not a secondary element but a key factor explaining both vulnerability and persistence of these behaviors across different population groups.

Regarding the predictive role of cognitive distortions, the logistic regression model showed that both gambler’s fallacy and illusion of control significantly predict the likelihood of exhibiting problematic behaviors (OR = 1.21–1.24) The model explained 38% of the variance and showed good discriminative capacity (AUC = 0.81), indicating that these variables are key predictors of gambling risk among university students with motor disabilities. These results align with a broad body of research identifying erroneous beliefs about chance and control as key factors in the development and maintenance of problem gambling (Samaniego et al., 2025; Solé et al., 2023). The literature indicates that cognitive distortions are not merely correlates but function as important risk factors that can anticipate the severity of gambling behavior across different contexts (Ahluwalia et al., 2026; Oyanedel et al., 2024; Vepsäläinen et al., 2024; Xu et al., 2025).

In this direction, Chan et al. (2019), in their review, concluded that cognitive distortions, including gambler’s fallacy and illusion of control, are central components in the maintenance schemas of pathological gamblers and represent relevant targets for therapeutic intervention, as they consistently predict gambling severity and the persistence of problematic behaviors. Studies specifically focused on online gambling have found similar results: Vepsäläinen et al. (2024), and more recently Xu et al. (2025), reported that distortions related to online gambling are significantly associated with problem severity and can be used to distinguish between gamblers with different levels of severity.

Furthermore, more recent studies have observed that higher scores on measures of cognitive distortions are associated with a greater likelihood of being classified as a problem or at-risk gambler, both in clinical populations and community samples (Mide et al., 2023; Peter et al., 2020; Ubillos-Landa et al., 2024). These studies support the idea that cognitive distortions not only explain associations with problematic behaviors but may also have incremental predictive value in statistical models, which is consistent with our logistic regression findings.

From a practical perspective, the accumulated evidence suggests that including measures of cognitive distortions in clinical assessments and prevention programs can significantly improve the identification of at-risk individuals before they develop severe gambling problems. In this regard, the graphical representation of the model and the ROC curve obtained reinforce its clinical and preventive utility, offering a quantitative tool that allows differentiation between students with and without risk based on measurable cognitive patterns.

Therefore, the results of the present study, as well as those of previous research, support that cognitive distortions are reliable predictors of gambling risk, emphasizing their normative and theoretical relevance both for understanding underlying psychological mechanisms and for guiding intervention strategies based on modifying erroneous beliefs.

Despite the relevance and consistency of the findings, this study has several limitations that should be considered when interpreting the results. First, the cross-sectional design prevents establishing causal relationships between online gambling participation, cognitive distortions, and gambling risk, making it impossible to determine whether cognitive distortions are a cause or a consequence of problematic behavior. Second, the sample size and representativeness constitute a limitation: the study included 135 university students with motor disabilities, which restricts the generalizability of the findings to other universities, students with different types of disabilities, or different cultural contexts. Additionally, the data were based on self-report questionnaires, which may introduce biases related to memory, self-perception, or social desirability, potentially affecting the accuracy of the measures. Moreover, relevant variables were not controlled, such as anxiety, depression, socioeconomic conditions, or social support, which could influence the relationship between cognitive distortions and problematic gambling behavior. Finally, the lack of longitudinal follow-up limits understanding of the temporal evolution of gambling risk and the persistence of cognitive distortions, preventing the evaluation of changes over time and the effectiveness of potential preventive interventions. These limitations highlight the need for future studies to address these issues to consolidate and extend current findings.

Regarding future directions, it is recommended to conduct longitudinal studies to assess the evolution of cognitive distortions and gambling severity over time, as well as to explore potential mediators and moderators, such as academic stress, loneliness, or social support, which could influence the relationship between gambling participation and the risk of problematic behavior. It would also be useful to conduct comparisons across different types of disabilities to develop tailored and specific interventions. Finally, the implementation of preventive strategies focused on correcting cognitive distortions could be an effective approach to reducing vulnerability among university students (Suriá-Martínez et al., 2024a,b).

Based on these results, the findings of this study indicate that participation in online gambling is frequent among university students with motor disabilities, with sports betting and online casino gambling being the most prevalent modalities and those associated with higher risk profiles. Although most students were classified as non-risk gamblers, a significant subgroup showed risk or presented gambling problems, highlighting the need for early identification of the most vulnerable individuals. Cognitive distortions, specifically gambler’s fallacy and illusion of control, increased progressively with risk level and were significantly associated with the severity of gambling behaviors, confirming their central role as cognitive mechanisms that sustain and intensify problematic behaviors. Moreover, predictive analyses showed that these distortions are significant predictors of gambling risk, with a model capable of adequately discriminating between students with and without risk. These findings provide relevant evidence for the design of prevention and intervention programs, suggesting that strategies aimed at identifying and correcting cognitive distortions may reduce the likelihood of developing problematic behaviors in this population. Finally, the results highlight the importance of considering both gambling modality and individual cognitive factors when addressing gambling risk among university students with motor disabilities, providing a solid basis for tailored and targeted interventions, as well as for future longitudinal research aimed at understanding the evolution of these behaviors and optimizing preventive and clinical strategies.

## Data Availability

The raw data supporting the conclusions of this article will be made available by the authors, without undue reservation.
